# Anesthesia for the Repair of Coarctation of Aorta and Ventricular Septal Defect in a Patient With Heterotaxy Polysplenia Syndrome

**DOI:** 10.7759/cureus.70886

**Published:** 2024-10-05

**Authors:** Vipul Sharma, Sonalika Tudimilla, Mounika Yerramshetty

**Affiliations:** 1 Anesthesiology, Dr. D.Y. Patil Medical College, Hospital and Research Centre, Dr. D.Y. Patil Vidyapeeth (Deemed to be University), Pune, IND

**Keywords:** coarctation of the aorta, heterotaxy syndrome (hs), left-sided isomerism, situs inversus with levocardia, ventricular septal defect (vsd)

## Abstract

Coarctation of the aorta (CoA) accounts for a small percentage of all congenital heart diseases (CHD) and occurs with a rare incidence in live births. It is a frequently diagnosed cardiac defect in infancy, though some patients present later with severe complications and reduced life expectancy. Heterotaxy syndrome is marked by abnormal lateralization of abdominal and thoracic organs, including the cardiac atria. Cardiovascular anomalies are the primary cause of morbidity in children with heterotaxy syndrome. Early suspicion and accurate diagnosis enable a more focused and effective approach to treatment. This case report seeks to review the literature on this rare and remarkable subset of developmental anomalies to inform the reader about the various modes of presentation, clinical manifestations, and surgical and anesthetic management. Here, we present a unique case of a 14-year-old male with left isomerism scheduled for CoA and ventricular septal defect (VSD) repair. The following case report was previously presented at the World Conference of Anesthesia in March 2024, Singapore as a poster presentation.

## Introduction

Heterotaxy (HTX) is a genetic disorder involving an unusual alignment of thoracoabdominal organs along the left-right axis of the body, with an incidence of 0.81 per 10,000 births [1]. Patients with HTX often exhibit lungs with two lobes on each side, bronchi positioned below the arteries, and several spleens (polysplenia). Unusual cardiac positioning, atrial appendage morphology, dual superior vena cava (SVC), and interrupted inferior vena cava (IVC) along with the continuation of azygous and partial anomalous pulmonary venous connection (PAPVC) are observed in over 50% of cases associated with left isomerism. The sinus node is often absent, hypoplastic, or positioned abnormally, which can manifest as slow atrial or junctional rhythm, abnormal P-wave axis on electrocardiogram (ECG), or in some neonates with left atrial isomerism complete heart block can occur. The liver spans from the left hypochondrium to the medial aspect of the right hypochondrium [2-4]. The main physiology behind coarctation of the aorta (CoA) involves a narrowing or constriction of the aorta, typically just distal to the origin of the left subclavian artery, near the ductus arteriosus. This narrowing creates a significant pressure gradient between the upper and lower parts of the body. As a result, blood pressure is elevated in the arteries supplying the upper body (head, neck, and arms) and reduced in the lower extremities. The left ventricle pumps blood through the constricted area, leading to left ventricular hypertrophy over time. Reduced perfusion to the lower body can also cause symptoms like leg fatigue and poor organ function, while increased pressure in the upper body can lead to complications such as aneurysms or cerebral hemorrhages. Preoperative management aims to stabilize patients, control hypertension, manage heart failure, and optimize cardiac function. Intraoperatively, dual-site arterial monitoring is essential for assessing pressure gradients, and careful control of blood pressure is needed to avoid complications. Postoperative strategies focus on managing rebound hypertension, monitoring for residual or recurrent coarctation, and addressing potential complications such as spinal cord ischemia and respiratory difficulties. Long-term follow-up is crucial to detect late complications. In this case, we primarily focussed on maintaining intraoperative cardiovascular stability and ensuring good postoperative care to reduce morbidity.

## Case presentation

**Figure 1:**
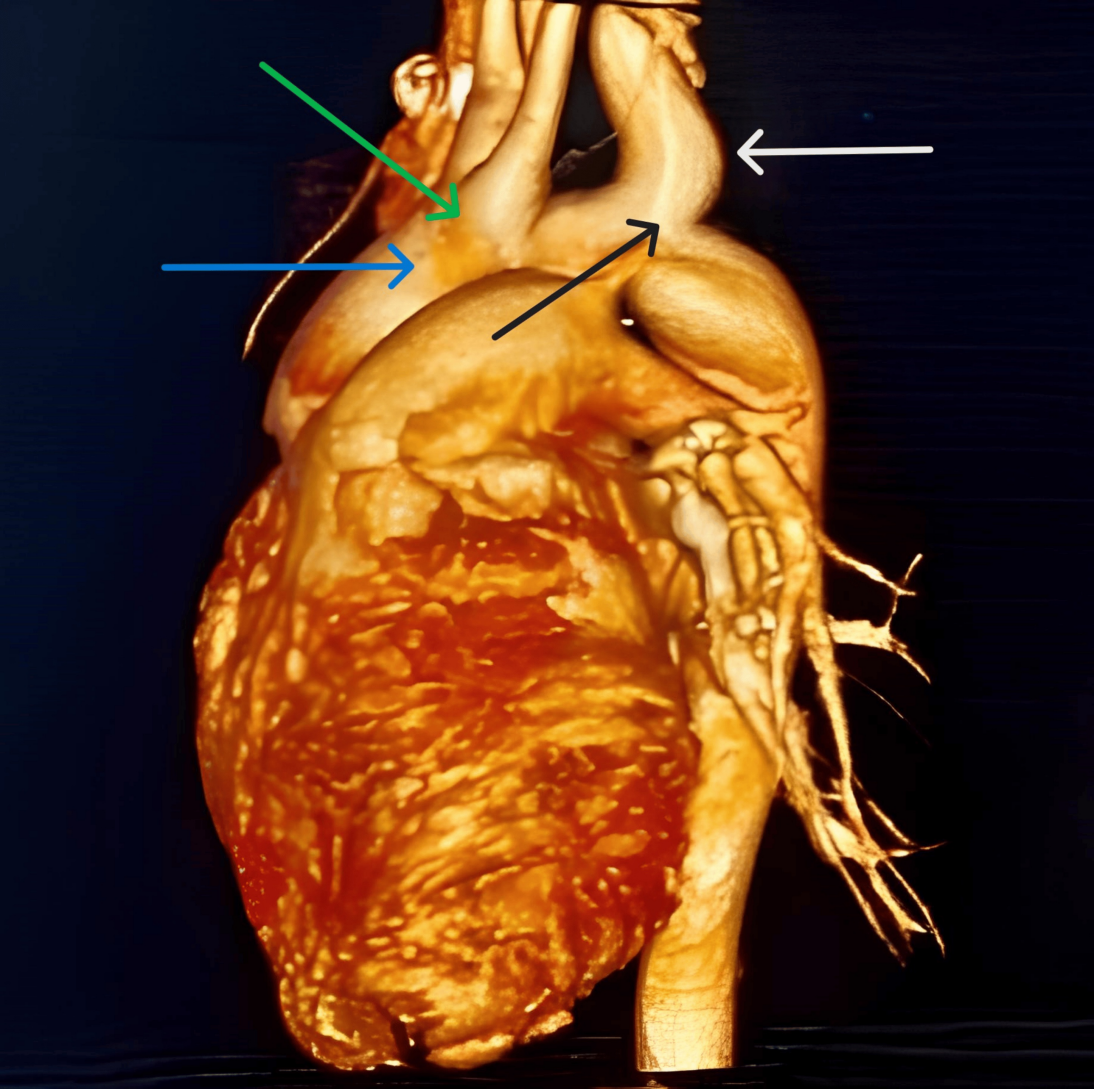
Computed tomography (CT) aortogram showing arch of aorta (blue arrow), common origin of right brachiocephalic and left common carotid artery (green arrow), aneurysmal dilatation of left subclavian artery (white arrow), coarctation of aorta (black arrow).

A 14-year-old male weighing 45 kg presented with hemoptysis (two to three episodes per day for three days), shortness of breath, and pulsatile headache with tinnitus. He had no complaints of loss of consciousness, syncope, fever, cold, cough, or loose motions. Systemic examination revealed a left parasternal heave, palpable P2, and a systolic thrill. Blood pressure was 142/94 mmHg with normal vitals. Chest X-ray showed levocardia with situs inversus. The ECG indicated a right bundle branch block. Echocardiography suggested situs inversus, levocardia, mid-muscular ventricular septal defect (VSD), moderate-sized patent ductus arteriosus (PDA), CoA, and mild-moderate pulmonary artery hypertension (PAH). To rule out an aortoesophageal fistula, an upper gastrointestinal endoscopy was performed, which was normal. A cardiac computed tomography (CT) aortogram showed a left-sided aortic arch with a shared origin of the left common carotid arteries and right brachiocephalic, aneurysmal dilatation of the left subclavian artery from its origin at the aortic arch, dual SVC, interrupted infra hepatic IVC, and suprahepatic IVC draining into the right atrium (Figure 1).

**Figure 2:**
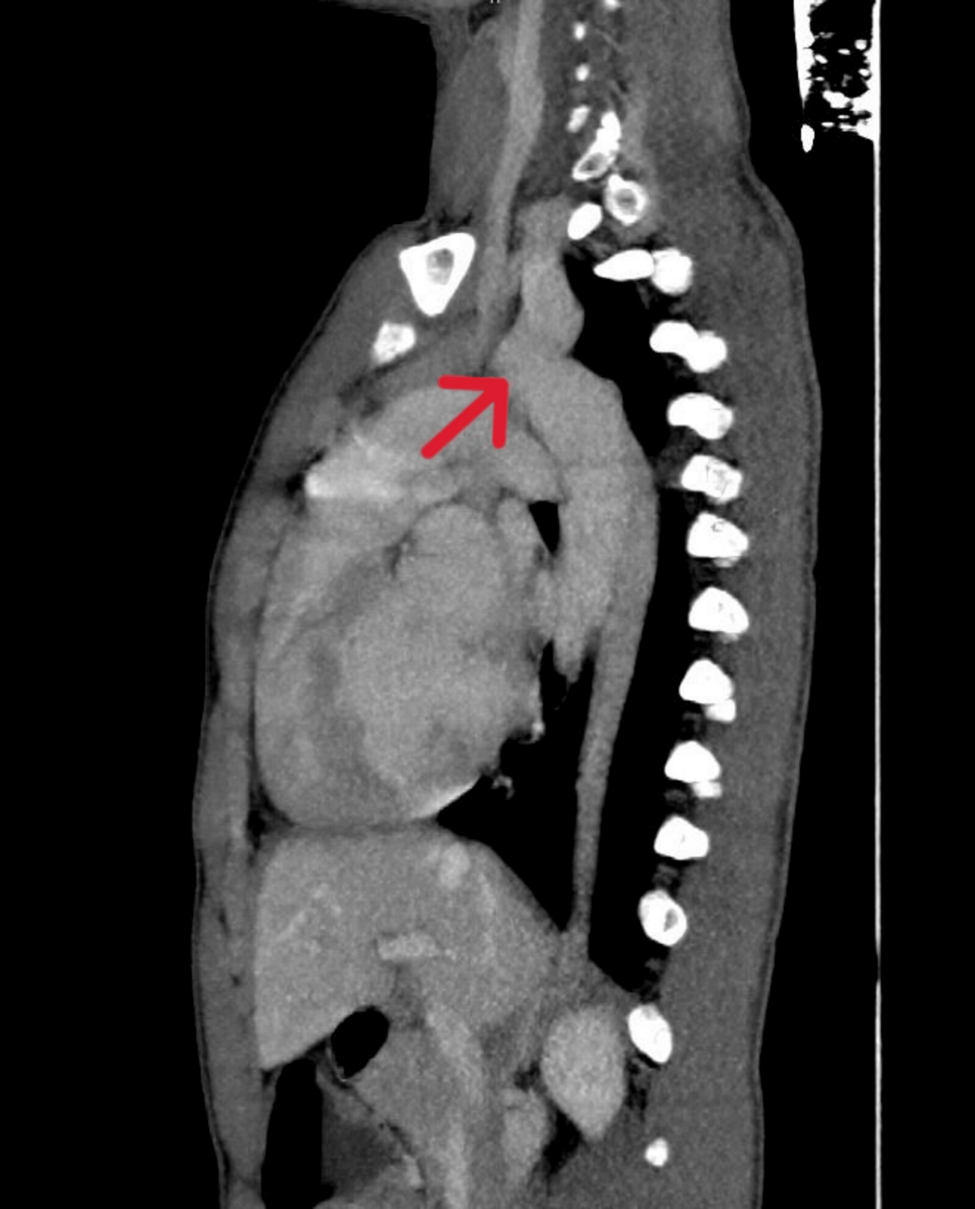
Computed tomography of chest showing the coarctation of aorta (red arrow).

**Figure 3:**
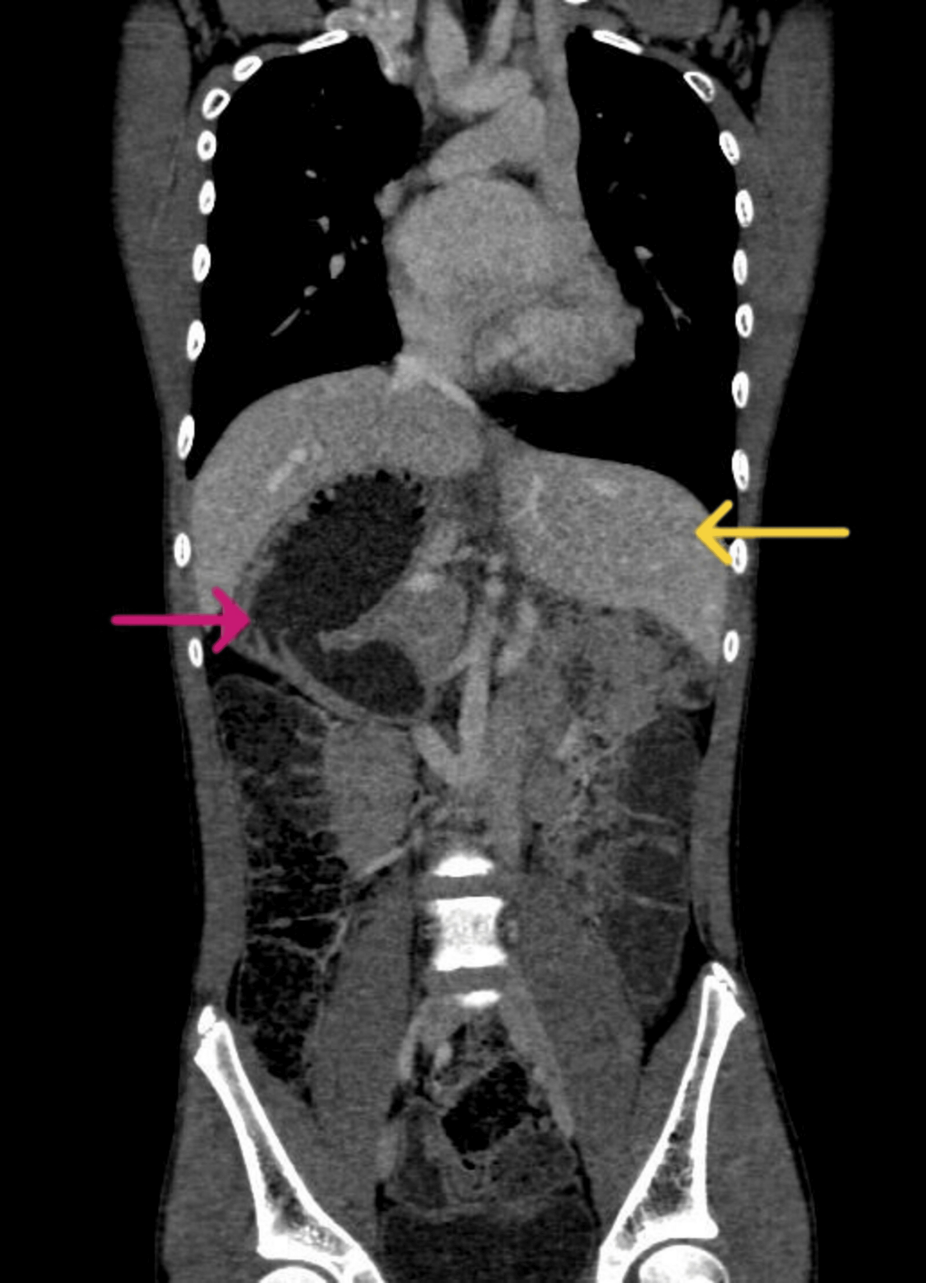
Computed tomography of the abdomen (coronal view) showing situs inversus with liver in the left hypochondrium (yellow arrow) and right-sided stomach (pink arrow).

Computed tomography revealed the CoA (Figure 2) and showed the liver in the left hypochondrium, polysplenia, right-sided stomach, and malrotated kidney (Figure 3). The patient was electively scheduled for CoA and VSD repair. Written consent was obtained. The patient had a one-phase repair for CoA and ventricular septal defect (VSD). Fasting status (Nil Per Os) was verified and the patient was taken inside the operation theatre, where all the standard monitors were attached. The patient was preoxygenated and Inj. midazolam (0.02 mg/kg) was given. Analgesia was provided with fentanyl (2 µg/kg). Induction was achieved using ketamine (2 mg/kg) and muscle relaxation was facilitated with vecuronium (0.1 mg/kg). A laryngoscopy was done and an endotracheal tube (size 6.5 mm internal diameter) was successfully placed. The patient was ventilated using isoflurane and a mixture of oxygen and air in a ratio of 1:1. Post-induction right radial and femoral arterial cannulation for continuous blood pressure monitoring was done. A subclavian central venous pressure (CVP) line was placed for inotropes administration and CVP monitoring. A thoracic epidural catheter was inserted to manage surgical stress and postoperative analgesia. Intraoperatively, an infusion of 0.2% ropivacaine with 2 µg/cc fentanyl was administered at 3 ml/hr. Infusions of nitroglycerine (0.5 mcg/kg/min) and dopamine (5 mcg/kg/min) were started and continued in the postoperative period. Cardiopulmonary bypass (CPB) was established through direct aortic cannulation, with moderate hypothermia (28ºC). To ensure brain protection, the aortic cannula was advanced into the innominate artery, and the brachiocephalic vessels were snared for regional cerebral perfusion.

Adequate anticoagulation was achieved with a heparin bolus of 300 U/kg, ensuring an activated clotting time (ACT) of over 480 seconds. Following CPB initiation, aortic cross-clamping was performed to create a bloodless surgical field. The total cross-clamp time was 25 minutes. The coarctation and patent ductus arteriosus (PDA) were repaired using an end-to-end anastomosis of the aorta. After the aortic arch was reconstructed, the VSD was closed with bovine pericardial patches [5] and interrupted pledget sutures during the rewarming phase. The patient was successfully weaned off cardiopulmonary bypass on the first attempt with minimal inotropic support. The total CPB time was approximately 50 minutes. Urine output, blood loss, and serial blood gas analysis were monitored. Post op recovery was uneventful and the patient was tapered off from inotropes gradually and extubated on the first postoperative day. He was comfortable and pain-free.

## Discussion

Anesthesia considerations

Successful management of patients with HTX requires an in-depth understanding of their unique anatomy. A thorough assessment by a pediatric cardiologist is necessary for all children diagnosed with HTX. In heart transplantation, cardiac anatomy can range from intricate single-ventricle congenital heart defects to a fully or almost fully functioning biventricular circulation. Given the mirrored position of the thoracic viscera, careful consideration is needed when placing a central line. The left internal jugular vein approach may be more prudent [4]. Ultrasound guidance should be utilized. Total pulmonary venous anomaly and right SVC are reported to accompany situs inversus, making the right internal jugular vein the preferable site for central venous cannulation [6]. Cardiovascular magnetic resonance imaging (CMR) is especially valuable in heart transplantation (HTX) for assessing complex intracardiac anatomy before surgery, as well as for evaluating older patients who often have suboptimal echocardiographic windows. CMR enables precise measurement of ventricular capacity, performance, and circulation, along with providing a detailed three-dimensional view of the anatomy. However, CMR has limitations, including the requirement for general anesthesia in younger patients, the risk of renal damage from gadolinium-based contrast agents, and the unsuitability for use with pacemakers [7].

Surgical considerations

In early infancy, cardiac surgery is determined by the intensity of symptoms, while in children beyond the age group of one year, the goal is to achieve stable single-ventricle or biventricular physiology. The primary reasons for surgical or catheter intervention in early infancy are obstructed total anomalous pulmonary venous return (TAPVR) and significant cyanosis caused by pulmonary sub-valvular stenosis for which balloon pulmonary valvotomy is done and is frequently linked to right isomerism [7]. Urgent pacemaker placement is required postnatally for certain neonates diagnosed with multiple spleens and complete atrioventricular (AV) dissociation. A partial cavo-pulmonary (Glenn) procedure is typically performed between the ages of three and six months. In this procedure, the single or bilateral SVC is attached to the pulmonary artery branch, allowing passive spontaneous circulation from the upper trunk into the pulmonary vascular system. In patients with an interrupted inferior vena cava (IVC), the azygos vein directs blood from the lower trunk to the SVC, further facilitating passive circulation into the pulmonary vascular system. This procedure is followed by the Fontan operation, typically performed around the age of five to 10 years, which involves linking the IVC to the appropriate branch of the pulmonary artery, completing the total cavopulmonary connection [8]. Two-ventricle repair has typically been performed in children who have two fully developed ventricles and distinct inlet valves. Staged ventricular recruitment, through atrial septal defect (ASD) restriction without closing the VSD, has been demonstrated to promote the growth of mildly hypoplastic ventricles and support eventual conversion to a two-ventricle system [9]. Patients with HTX are predisposed to multiple airway anomalies and infections (such as *Streptococcus pneumoniae* and *Hemophilus influenzae*) [10]. Pulmonary percussive therapy, bronchodilators, incentive spirometry, postural drainage, steroids, and antibiotics can optimize the pulmonary system before surgery. Premedication drugs that depress ventilation or ciliary activity should be avoided in patients with Kartagener syndrome associated with situs inversus. Nasal airways and/or nasal intubation should also be avoided due to sinusitis.

## Conclusions

Heterotaxy syndrome is usually linked with intricate cardiovascular abnormalities. Administering optimal anesthesia for such high-risk patients mandates a comprehensive understanding of the patient's anatomical and physiological intricacies, coupled with an awareness of the potential for hemodynamic instability to precipitate multi-organ dysfunction. Proper perioperative planning for coarctation of the aorta (CoA) involves key considerations such as patient age, the severity of the coarctation, associated cardiac anomalies, and preoperative hemodynamics. Neonates and infants may require urgent intervention, while older patients may benefit from stenting or more complex repairs. Preoperative imaging (echocardiography, computed tomography) guides surgical planning, and careful anesthesia, blood pressure, and fluid management are essential. Postoperative risks, including recoarctation and hypertension, necessitate extended ICU care and long-term follow-up to ensure optimal outcomes are crucial for successful outcomes in these patients.
